# Complete Genome Sequence of an Evolved *Thermotoga maritima* Isolate

**DOI:** 10.1128/genomeA.00557-15

**Published:** 2015-05-28

**Authors:** Raghuveer Singh, Julien Gradnigo, Derrick White, Anna Lipzen, Joel Martin, Wendy Schackwitz, Etsuko Moriyama, Paul Blum

**Affiliations:** a School of Biological Sciences, University of Nebraska–Lincoln, Lincoln, Nebraska, USA; b U.S. Department of Energy-Joint Genome Institute, Walnut Creek, California, USA

## Abstract

*Thermotoga maritima* is a hyperthermophilic bacterium with a small genome (1.86 Mbp). Genome resequencing of Tma200, a derivative produced by experimental microbial evolution, revealed the occurrence of deletions and substitution mutations. Their identification contributes to a better understanding of genome instability in this organism.

## GENOME ANNOUNCEMENT

*Thermotoga maritima* MSB8 genomovar DSM3109 is a hyperthermophilic anaerobic bacterium (1) that grows at 80°C, producing a maximum of 4 mol of H_2_ per mol of glucose (2). There are a variety of duplicated genes and direct repeats in its genome, suggesting the potential for genome instability. Genome resequencing of *T. maritima* MSB8 genomovar DSM3109 in 2011 and 2013 (3, 4) indicated that the earlier sequenced *T. maritima* MSB8 reported by Nelson et al. (NC_000853.1) (5) was an evolved laboratory variant with an approximately 8-kb deletion located between TM1847 and TM1848 (annotation according to reference 5). The 8-kb deletion may have resulted from genome instability. To assess the potential for additional instability, a cell line harboring a chromosomally integrated kanamycin-resistant suicide plasmid was allowed to segregate without drug addition but with selection for maltose catabolism as part of ongoing studies involving experimental microbial evolution (unpublished data). The initially sequenced genome of *T. maritima* by Nelson et al. (5) (NC_000853.1) was used to describe the genome changes in the resulting strains. Of 50 clonal isolates screened, 10 underwent deletion formation, including a 10-kb loss between TM1322 and TM1332. One of these 10-kb deletion isolates was named Tma200. The deleted region in Tma200 encoded five hypothetical proteins, two AstB/ChuR-related proteins, one LacI family transcriptional regulator, and three ABC transporter ATP-binding proteins. In addition, two distinct repeat sequences of 921 bp and 313 bp, respectively, were identified in TM1322 (coordinates 1340942 to 1341862 and 1342246 to 1342558) and TM1332 (1350970 to 1351890 and 1352274 to 1352586). Crossover between the 921-bp direct repeats deleted the intervening region (1341863 to 1351890). Genome resequencing of this *T. maritima* derivative expands the understanding of factors underlying genome instability in this lineage.

Genomic DNA was isolated from Tma200 as described previously (6). A DNA library was prepared from ~500-bp fragments of randomly sheared genomic DNA. This library was sequenced using an Illumina HiSeq 2000 sequencer and generated 100-bp paired-end reads. FASTQ files containing the short reads were mapped to the most recent reference genome of *T. maritima* reported by Latif et al. (4) (NC_021214.1) using Bowtie 2 version 2.1.0 and IGV version 2.3 to locate mutations and deletions that were then verified by DNA sequencing of PCR amplicons. A full consensus genome (1,859,582 bp) was generated using SAMtools version 1.1 and BCFtools alignment processing utilities version 1.1 (7) with the *T. maritima* NC_021214.1 as a reference.

The genome was annotated using the NCBI Prokaryotic Genome Annotation Pipeline (http://www.ncbi.nlm.nih.gov/genome/annotation_prok). This pipeline identified 1,918 genes, 1,862 coding DNA sequence (CDS), 6 pseudogenes, 3 rRNAs, 46 tRNAs, and 7 clustered regularly interspaced short palindromic repeat (CRISPR) clusters.

### Nucleotide accession number. 

The complete genome sequence has been deposited in GenBank under the accession number CP010967.
